# Is Early Experience Destiny? Review of Research on Long-Term Outcomes following International Adoption with Special Reference to the British Chinese Adoption Study

**DOI:** 10.1155/2016/6303490

**Published:** 2016-05-10

**Authors:** Margaret Grant, Alan Rushton, John Simmonds

**Affiliations:** ^1^CoramBAAF Academy of Adoption and Fostering, London WC1N 1AZ, UK; ^2^Institute of Psychiatry, Psychology and Neuroscience, King's College London, London SE5 8AF, UK

## Abstract

The pathway from adverse early experience to adulthood for internationally adopted children is complex in identifying key influences, impacts, and outcomes. This review arose from the authors' involvement in the British Chinese Adoption Study, a recent outcomes study that explored the links between early orphanage care, adoptive experiences, and midadulthood. It differs from previous reviews in focusing on a greater length of time since adoption. Both quantitative and qualitative studies were included to allow for examination of a fuller range of adult-related outcomes rather than mental health scores alone. The sampling, methods, and results of reviewed articles are summarised and a critical commentary is provided. Despite methodological differences and identified strengths and weaknesses, conclusions are drawn on the basis of the evidence available. Special attention is paid to the interpretation of negative outcomes. Findings identify areas that should be explored further in order to gain a fuller understanding of midlife outcomes of people who experienced a poor start in life followed by international adoption. Such studies help in refining lifespan developmental theories.

## 1. Introduction

A substantial body of literature has developed which identifies the adverse impact on children's development over time when their birth families cannot provide them with sensitive, reliable, and committed care. This is particularly stark for children who are separated from their birth families, including those who go on to be internationally adopted. Freud was hugely influential in articulating a powerful conceptual model of human development that came to influence thinking over a generation and more. He noted the long-lasting impact of adverse early experiences on the “unconscious mind”:* “We must assume… that the very impressions which we have forgotten have nevertheless left the deepest traces in our psychic life, and acted as determinants for our whole future development”* [1]. Subsequent developments have been equally significant, particularly those of Bowlby and his concept of attachment. This is again summed up by a particularly strong and influential statement:* “Prolonged deprivation of the young child of maternal care may have grave and far reaching effects in his character and so on the whole of his future life. ‘Good mothering' was almost useless if it was not available in the first few years of life”* [2]. Bowlby came to significantly modify this view when he said that human development* “turns at each and every stage of the journey on an interaction between the organism as it has developed up to that moment and the environment in which it then finds itself” *[3].

The scientific community has developed the research base for further understanding the significance of early experiences, and this is set out in great detail, particularly in neuroscience. But there is still a challenge in establishing a clarity of view of the influence of early experience on later development and then throughout the life course.

The authors' interest in this topic arose from the experience of conducting the British Chinese Adoption Study, based on data collected from 72 women in their 40s and 50s who had been adopted from Hong Kong to the UK in the 1960s and 70s. The study explored the links between early institutional care, international adoption, and subsequent experiences and midlife outcomes. Access to contemporaneous data from the records offered a relatively rare opportunity for matching individual data recorded on the women's early lives with self-reported information gathered around 45 years later. From the original group of 100 girls, 99 adult women were traced and 72 agreed to participate in the research study [4]. The hypothesis to be tested was that adverse early experiences would result in poorer outcomes for this group compared to age-matched UK peers. Results from this study have been reported [4–6] and are included alongside the findings from other studies discussed here.

In that context, this paper aims to review the existing research literature on international adoption long-term outcomes with a particular focus on midlife. The main midlife adult studies are presented with details of samples, methods, measures, and conclusions. The discussion concentrates on the issue of contrasting outcomes and the factors that may influence diverse results. We note that complementary reviews of international adoption literature have been completed, for example, on childhood outcomes [7], behavioural problems in adolescence [8], the experiences of South Korean-born adopted people [9], counselling for members of the adoption triad [10], and links between adopted people's adjustment and their adoptive parents' cultural competency [11]. To our knowledge, this is the first review to focus on outcomes in midlife and examine internationally adopted adults' lives over a period of several decades. 

*International Adoption: Risks and Recovery in Childhood and Adolescence.* The majority of evidence to date comes from studies of internationally adopted children to adolescence and young adulthood. Many of these cohorts spent their early lives in orphanage care: a specific form of early adversity in circumstances that are unlikely to provide children with the personalised, responsive care associated with optimal development. While in institutional care, children typically display delayed physical growth, cognitive development, and behavioural development, on average 1 SD below expected levels for noninstitutionalised children's development in each of these domains [12]. Across domains, risks are elevated for children who have experienced either longer exposure to orphanage care or very poor quality of care [13]. Understanding how quality of care relates to later outcomes is often complicated by lack of individualised data about children's orphanage care experiences and their individual responses to the care they receive.

As well as risks, studies have produced evidence of developmental recovery across domains, sustained over periods of several years, for ex-institutional children after they have been placed for adoption. Not all children with longer exposure to very poor quality care have poorer outcomes, and the level of individual problems differs substantially within groups [14]. In all cohorts, some children are found to recover more quickly than others and to a greater extent [13, 15, 16]. Consistent with evidence from domestic adoption, the age at which children join their adoptive family is associated with patterns of subsequent recovery. At a group level, younger children fare better, but the relationship between age at adoption and subsequent developmental recovery is not consistent across studies [12, 17, 18].

Many, although not all, cases of international adoption involve children being raised in families who do not reflect or share the variety of factors that make up a child's heritage. Whether families can enable their transracially adopted children to develop a positive, cohesive sense of self and identity remains a closely scrutinised issue in both domestic and international adoptions [19]. The specific experiences that can arise from being transracially adopted have been referred to as “disjointedness” [20] or the “transracial adoption paradox” [21] and much practice effort has focused on the process of making links with birth culture or “reculturation” [22]. Research on the relationship between ethnic identity and psychological adjustment for transracially adopted cohorts (both domestic and international) has proved inconsistent, with some studies identifying significant associations and others not [23].

Several adolescent/young adult studies have found that comparably raised risks for severe outcomes (e.g., suicide, suicide attempts, and psychiatric admissions) were associated with older age at adoption among other factors [24]. Although the remit of this paper is limited to later stages of adulthood, we note the growing number of studies involving young adult cohorts.

A number of methodological challenges exist in understanding the effects of early experiences on the lives of internationally adopted people. The possible influences are numerous and vary greatly across cohorts: genetic and perinatal factors (often undocumented/unknown); duration and quality of preadoption care; postadoption environmental variables [7]. In particular, data on preadoption experiences may be inconsistent or only partially recorded. Identifying which components of early care predict which outcomes, or whether there is an overall effect detectable across several domains, is extremely challenging [13]. The contribution of the study of epigenetic factors offers much potential but has yet to become a source of definitive answers, particularly in development across the lifespan.

*Current Paper: Review of Midadulthood Studies.* While gaps remain, the literature so far points to patterns of both risks and recovery in childhood and adolescence. A longer-term perspective is needed to understand how people's lives unfold as they leave their adoptive family homes and establish relationships with partners, friendships in adulthood, and identities as parents, students, workers, or even, following parental death, the oldest generation of their family. Core to this paper is the following question: by midadulthood, do significant risks persist or does recovery continue?

## 2. Method

An initial list of studies was collated based on sources we had consulted in planning the British Chinese Adoption Study. A subsequent search was carried out on the PsycINFO internet-based bibliographical database with additional hand-searching of key journals.

Although we were interested in ex-orphanage samples, studies of internationally adopted adults with mixed or unknown early experiences were included due to limited number of exclusively ex-orphanage cohorts. This allowed us to gain a broader picture of potential influences across the life course of internationally adopted adults. Cohort age ranges varied substantially, and many studies included some adults in midlife along with younger counterparts. Because of our focus on midlife, we included only those studies with a mean participant age of more than 25 years (in order to exclude studies of emerging adulthood, commonly defined as the period between 18 and 25 years). If mean age was not specified, the decision was based on whether the age range and reported findings suggested that it was likely that the sample would meet this criterion. Studies with very large age ranges where results were not disaggregated to show midlife outcomes were excluded.

For the PsycINFO database search, the following search terms were entered: [adopt^*∗*^ AND intercountry (all text)]; [adopt^*∗*^ AND orphanage (all text)]; [adopt^*∗*^ AND institutional care (all text)]; [adopt^*∗*^ AND ex-institutional (all text)] [adopt^*∗*^ and international (abstract)]. Terms such as “adulthood” and “adult” were not used due to previous experience of finding that entering these terms resulted in papers being overlooked.

These searches returned a combined total of 420 abstracts. Abstracts were then viewed individually and screened using the following criteria.

### 2.1. Inclusion Criteria

Inclusion criteria are the following:

1. Investigated outcome or experiences of internationally adopted adults.
2. Mean age of sample over 25 years.
3. Published in peer-reviewed journal.

### 2.2. Exclusion Criteria

Exclusion criteria are the following:

1. Full text not published in English.
2. Nonempirical studies.

After initial screening, 66 papers were identified that potentially or clearly fitted our criteria. Full text articles were accessed and assessed for eligibility. Main reasons for rejection were as follows: (a) age of sample not fitting criteria, (b) nonrelevancy to topic (e.g., “adoption” referred to adoption of a new policy or intervention, not to adoption of children), (c) full text not published in English, or (d) nonempirical papers. A total of 19 papers, which reported findings from 11 studies, were identified for inclusion.

A core concern is the extent to which evidence has so far accumulated on whether risks associated with early preadoption experiences and subsequent recovery continue into midlife for internationally adopted adults. In addition, we explore potential modifying influences that emerge over time, such as identity and adjustment processes related to being internationally adopted. Each of the papers included for this review contributes to these aims.

## 3. Results and Discussion

To aid interpretation of results, studies that test across-group differences for outcomes are presented separately from single-cohort studies.  Table 1 shows six studies that include comparison groups, with results reported in 13 papers.  Table 2 includes five studies (six papers) using qualitative or mixed methods approaches for within-group analysis only.

Results related to the same cohort are grouped together: seven papers for a large Dutch cross-sectional study [25–31]; two papers for the British Chinese Adoption Study [5, 6]; and two papers for a qualitative study from the US [32, 33].

The review is structured in three main sections. First, adult outcomes are reported for psychological adjustment and social functioning/relationships. Second, predictor variables from early life are explored. Third, findings on social location, including specific experiences arising from being internationally adopted adults, are examined.

### 3.1. Adult Outcomes

#### 3.1.1. Psychological Adjustment

Only four studies included across-group comparisons based on standardised mental health measures or indicators. Previous studies with younger adult cohorts (up to mean age of 25 years), particularly those based on epidemiological data from Swedish national datasets, indicated raised risks for serious mental health problems among internationally adopted adults [34]. Exploring whether such results were replicated in the limited number of midlife adult cohorts was central to this review.

von Borczyskowski et al. [35], in a Swedish study based on national health discharge and “cause of death” registers, found higher risks for suicide attempt and suicide death among internationally adopted adults (*n* = 6,065) than among either domestically adopted adults (*n* = 7,340) or Swedish-born general population (*n* = 1,269,318). The confidence intervals for these results are notably narrower than those in some studies with younger cohorts: suicide attempt, risk ratio 4.5 (95% CI 3.7–5.5); suicide death, risk ratio 3.6 (95% CI 2.6–5.2). The relative risk was found to be higher for women than for men. In other words, the gap between internationally adopted and nonadopted women was larger than the gap between the two male groups [35].

In a large Dutch study, Tieman et al. [25] found that internationally adopted adults from a range of countries of origin (*n* = 1,484; age range 24–30 years) were more likely than an age-matched general population sample to meet the criteria for an anxiety disorder or substance abuse or dependence. Adopted and nonadopted women were more likely to have an anxiety disorder than men; men were more likely to meet the criteria for a disruptive disorder or substance misuse. Adopted men, however, showed greater prevalence of mood disorders than nonadopted men. No such evidence was found when comparing the female groups. An earlier finding, based on data collected from the same cohort aged 14–18 years, had shown an increased risk for a disruptive order, but this was not replicated in the data from adulthood [25]. The availability of data collected from parents on mental health problems in childhood and adolescence, combined with data from the adopted people in adulthood, was a significant advantage in this study. A separate paper, as part of the same longitudinal study, compared biologically related and nonrelated adopted siblings and found that both adopted adult and adoptive parent reports indicated that genetic factors had greater influence on internalising problems, while environmental factors had more influence on externalising problems [28]. Subsequent papers reporting on the same Dutch sample explored the within-group links between early childhood adversities and adulthood anxiety and are reported in the next section.

Storsbergen et al.'s [36] smaller Dutch study compared outcomes for 53 adults adopted from orphanage care in Greece with nonadopted Dutch-born adults. The adopted men reported higher rates of depression than nonadopted men, but no other statistically significant differences in psychological adjustment between the adopted and nonadopted groups were found on the selected measures [36]. The authors note that their overall findings of “adequate adjustment” for the adopted adults as a group may be influenced by the relatively high quality of care in the Greek orphanage. Mean age at placement for adoption was also relatively young at 9 months (SD: 8.58; [36]).

The British Chinese Adoption Study also found that psychological adjustment was comparable for women adopted internationally from Hong Kong and their UK-born peers. Using standardised measures (General Health Questionnaire (GHQ12, [37]); Malaise Inventory [38]; Rosenberg Self-Esteem Questionnaire [39]) and an index of whether women had ever sought professional help for anxiety, depression, or other mental health problems, no significant differences were found on comparisons with either nonadopted women (*n* = 5,115) or domestically adopted women (*n* = 50) [5]. As with Storsbergen et al. [36], this suggests that where institutional care is not globally depriving and where adoptive families provide, for the most part, support and care for the remainder of childhood and adolescence, the timing and exposure of orphanage care do not necessarily predict midlife outcomes [5]. Average age of adoption in the BCAS sample (23 months) was older than that in the study of Storsbergen et al. [36]. Within-group tests with the women adopted from Hong Kong identified that retrospectively reported experiences of low care within the adoptive family and a negative view of their adoption were both associated with poorer current mental health [5].

The relatively limited numbers of studies of psychological adjustment in midlife preclude strong statements on the long-term relationship between orphanage care, international adoption, and psychological adjustment at this stage. In individual studies, results are heterogeneous. As with younger cohorts, enduring effects were found in some cohorts, although the extent, domains, and proportion affected varied. We note, however, that two studies found no evidence of raised risks for poor psychological adjustment in samples that experienced international adoption following orphanage care that was not severely depriving, suggesting the potential for recovery over the long term. Nevertheless, evidence from large-scale studies that found somewhat raised risks for severe mental health problems cannot be dismissed.

#### 3.1.2. Social Functioning and Relationships

Irhammar and Bengtsson [40] used the Adult Attachment Interview (AAI) [41] with a sample of 40 internationally adopted adults raised in Sweden (mean age, 28 years; 57% adopted after the age of 18 months). The AAI involves specialist interviews based on which researchers assign participants' attachment styles to predefined classifications: dismissive (inconsistent, contradictory reports of attachment-related relationships) or preoccupied (confused, angry, or passive preoccupation with attachment figures) or autonomous (balanced reports of attachment figures delivered coherently, consistently, and plausibly). Dismissive and preoccupied styles are categorised as insecure and autonomous styles as secure. In addition any interviews can also be categorised as unresolved in relation to previous traumatic experiences or loss.

Results for the internationally adopted adults in this Swedish study were classified as follows: secure (47.5%), preoccupied (25%), dismissing (27.5%), and unresolved (17.5%). In comparison to a female nonclinical group of mothers, although the proportions of adopted adults with dismissive or preoccupied classification were higher, differences were not statistically significant. Within-group analysis identified that factors including later age at adoption and a desire to know more about one's origins were significant predictors of insecure attachment styles in adulthood [40]. As the single study measuring adult attachment styles, and with a small sample size, caution should be exercised in extrapolating these results to all internationally adopted adults.

Tieman et al. [26] took a broader approach to measuring adult social functioning. They used a 115-item Dutch scale (Groningen Questionnaire about Social Behaviour), which includes subscales on family functioning and relationships with partners, parents, friends, and siblings, to compare internationally adopted adults (*n* = 1,417) to a general population sample (*n* = 713) [26]. Although the adopted group were less likely to be in a partnership, live with a partner, or be married, they reported better functioning without a partner than the comparison group. After adjusting for socioeconomic differences, the adopted group were as likely as their nonadopted peers to have children. Some indications of poorer functioning in relationships with parents and siblings were found in the adopted group, but also better functioning than nonadopted peers was found for functioning in friendships. The authors conclude that there was no evidence of widespread difficulties with social adjustment among the adopted group [26].

In the British Chinese Adoption Study, the internationally adopted women (*n* = 72) were no less likely than UK comparison groups (nonadopted *n* = 5,115; domestically adopted *n* = 50) to report having a confidant(e) they could talk to about worries or problems (over 97% in each group). Nevertheless, talking to family members or others about adoption-related experiences was difficult in some cases. Nonsignificant differences were found in the likelihood of being married or cohabiting. Relationships with adoptive parents were reported, in the main, to be functioning well or satisfactorily most of the time. Retrospective reports indicated change in both directions over the life course: relationships with parents and/or siblings could deteriorate or ameliorate over time [5].

In their smaller-scale qualitative studies, Docan-Morgan [33] and Lindblad and Signell [42] included questions about confiding in parents and others about experiences such as dealing with derogatory comments, hostile reactions, or other adversity in relation to visible ethnic minority status or adoptive status. Docan-Morgan [33] found that, among 34 Korean-born adopted adults in the US, some talked openly with their parents about “racial derogation” experiences and found it helpful, for example, when parents were able to discuss what they had experienced without minimising it, even if they could not suggest solutions. Others avoided raising the topic with their parents, either because they assumed their parents would be unwilling or unable to help or because they had experienced their parents' response in the past as unhelpful or dismissive [33]. Lindblad and Signell [42] also reported that, in their sample of internationally adopted women (*n* = 17), worries about their parents' feelings often prevented detailed discussions on these topics.

In summary, the evidence on social functioning and relationships is mixed, although studies so far do not suggest widespread difficulties at a group level. With limited studies to draw from, all using different measures or approaches, clear conclusions on social functioning and relationships among internationally adopted adults are not yet possible.

### 3.2. Exploring Early Life Predictors of Midlife Functioning: Preadoption Care, Age at Adoption, and Adoptive Family Environments

#### 3.2.1. Preadoption Care

Few studies of internationally adopted adults address the issue of preadoption experiences in depth, although, as noted earlier, childhood studies have identified poor quality of care and duration of orphanage care as potential predictors of later difficulties.

Only one adult longitudinal study [29] collected comprehensive data via parental reports on children's early care. This study asked adoptive parents to rate their children's preadoption experiences on a 3-point scale of adversity (none, somewhat, and severe) and also to report whether they were certain of these experiences. Only information reported as “certain” was included. At follow-up (aged between 22 and 32 years, *n* = 1,364), those participants who had experienced multiple early adversities (defined as a combination of physical neglect and/or abuse and multiple placements prior to adoption) were more likely to have mood or anxiety disorders or substance abuse/dependence in adulthood [29]. Comparing these results to the two earlier follow-ups of the same group, the researchers found that this pattern remained relatively stable over time. In other words, those adults with disorders in adulthood were more likely to have been reported by their adoptive parents as having mental health problems during adolescence. However, for some participants, disorders only emerged in adulthood [29].

In within-group analysis from the same study, the researchers examined the associations between parent-reported data on early experiences and cortisol levels among a subsample (*n* = 623) of the adopted adults, based on the hypotheses that extreme stress in early childhood may affect changes in the hypothalamic-pituitary-adrenocortical (HPA) axis. Saliva samples were collected four times over a single day. Lower morning cortisol levels were found for those who had experienced severe neglect or abuse compared to those who did not. Severe early neglect was associated with flatter diurnal curve; moderately severe early abuse was associated with high cortisol levels and a steeper cortisol diurnal slope. In other words, parent-reported early neglect and abuse appeared to predict alterations to cortisol levels among adopted adults several decades later, despite the experience of a substantial change of environment following early maltreatment [30]. The researchers subsequently identified that childhood adversity modified the relationship between adulthood anxiety disorders and cortisol secretion, but these results were not replicated for mood disorders [31].

Two studies had access to information about the specific orphanages that their samples had lived in prior to adoption [5, 36]. Storsbergen et al. [36] drew on contemporaneous observations from previous research and reported that the orphanage made efforts to limit the child : caregiver ratio (10–12 infants per 2-3 caregivers) and ensure consistency of caregivers. Only one significant difference in psychological adjustment (mental health, well-being, and self-esteem) was reported on comparisons to Dutch-born general population sample: adopted men were more likely to report problems with depression [36].

In the British Chinese Adoption Study, reports from Childcare Officers who visited the orphanages in Hong Kong were available to help categorise the early experiences of the women [5]. As children, they had been cared for in four orphanages which, when compared to available information on other institutions, seem to have provided a reasonable quality of basic care within the constraints of group care (ranging from 65 to 450 children depending on the orphanage). None of the orphanage-related variables that were tested (including a binary variable singling out the smallest orphanage as potentially providing a better standard of care) proved to predict midlife outcomes.

In the distinction between “globally depriving” and “psychosocially depriving” quality of care, both of these samples' early experiences would fit the latter category. Consistent, personalised attention from familiar caregivers was in short supply but physical care, adequate nutrition, and other basic needs appear to have been met. Other studies reported more briefly on the preadoption circumstances of their cohorts (see Tables 1 and 2) but did not use these as potential predictors of adult outcomes.

#### 3.2.2. Age at Placement

Evidence on whether later age at placement, as found in some studies of younger cohorts [7], continues to predict poorer outcomes into adulthood proved inconsistent in the studies reviewed here. The evidence base is particularly limited as age at adoption was not always explored because data were either not available or not tested as potential predictors. The only study to use adult attachment classifications as its outcome measure found that insecure attachment classification (at mean age of 28 years) was associated with later age at placement, although the sample was relatively small (*n* = 40) [40]. However, Tieman et al.'s [25] within-group tests for predictors of psychiatric diagnoses on 1,484 internationally adopted Dutch adults found no association with age at placement. Similarly, for the British Chinese Adoption Study, the hypothesis that age at adoption would predict midlife psychological adjustment was not supported [5]. Using a composite outcome index as the dependent variable of psychological adjustment, no evidence was found that age at adoption predicted later functioning [5].

#### 3.2.3. Adoptive Family Environments

In childhood studies, differences in adoptive family environments beyond broad characteristics usually remain unspecified, partly due to lack of variation on factors such as socioeconomic status or the use of measures too broad to pick up subtle yet potentially important differences [12, 43]. The evidence on family risk factors (such as parental mental health or marital discord) is also relatively sparse and results are inconsistent. However, parental warmth, sensitivity, and stimulation have been found to be associated with better outcomes [18].

In the studies reviewed here, data on adoptive family environments were also restricted, but some relevant findings did emerge. Tieman et al. [25] found some indication that adults adopted into higher socioeconomic status families might be more likely than those adopted into less affluent circumstances to have poorer outcomes. The authors speculated that families with higher socioeconomic status might place greater emphasis on academic and other achievements, but data to test this hypothesis further were unavailable [25].

In the British Chinese Adoption Study, two statistically significant associations were found between adoption-related experiences and adult outcomes. Those women who reported poor care, lacking in warmth, understanding, and/or acceptance [44], from both of their adoptive parents during childhood and adolescence also reported poorer psychological adjustment [5].

Overall, we are limited in the conclusions that can be drawn on early predictors. Although van der Vegt et al.'s [29, 30] cross-sectional sample, which identified an association between early adversity and later outcomes, was large (*n* = 1,364), the authors are appropriately cautious in concluding that this offers partial, rather than conclusive, evidence of persistent effects for children who had experienced a combination of early adversities, given the lack of studies to date to replicate such analysis. In addition, across studies, not all cohorts were purely ex-orphanage samples, and data on quality of orphanage care were absent in all but two studies, both of which relied on information collected for service delivery rather than research purposes [5, 36].

### 3.3. Social Location following International Adoption

#### 3.3.1. Adoption Appraisal and Adoptive Identity

A notable finding from the* Beyond Culture Camp* study was that being adopted became an increasingly important issue with age for both Korean-born and white US-born adopted adults [45]. This finding contradicts the assumption that the greatest difficulties will be faced when an adopted person enters adolescence and when adoption can add an additional layer of complexity to normal processes of identity development [45]. Higher self-esteem, gender (being female), and higher levels of life satisfaction were associated with feeling more comfortable with adoptive identity for the Korean-born adults [45]. By contrast, for the white domestically adopted participants, higher life satisfaction and living in a more ethnically homogenous area were associated with feeling more comfortable with adoption identity [45].

Two other studies specifically measured “adoption appraisal,” meaning whether adopted adults view their adoption experiences positively or negatively (in a global sense rather than in relation to specific experiences such as racial derogation). Storsbergen et al. [36] collected self-reported ratings of participants' adoption experiences on a 5-point Likert scale from very positive to very negative. The researchers predicted that a negative appraisal of adoption would be associated with poorer outcomes [36]. Initial tests showed that adoptees who either* had* searched or* were currently* searching for birth parents reported higher rates of difficulties: poorer mental health, well-being, and self-esteem. Subsequent analysis indicated that whether someone rated their adoption negatively (rather than neutrally or positively) was a better predictor of poor psychological adjustment than whether they had engaged in a search for their birth parents. A negative appraisal of adoption was associated significantly with both more mental health problems and less well-being [36].

The British Chinese Adoption Study included questions asking the women whether they felt happy about being adopted, loved in their family, and a sense of belonging [5]. As with Storsbergen et al. [36], those women who rated their adoption experiences negatively reported poorer psychological functioning than those who viewed their adoption experience as either positive or neutral.

#### 3.3.2. Ethnic/Cultural/Social Identities

In McGinnis et al. [45], the Korean-born adopted adults, as opposed to their white US-born counterparts, reported that race/ethnicity was important at all ages. By young adulthood, 81% agreed it was important. Self-esteem was a significant predictor of feeling comfortable with their racial/ethnic identity. Reponses to open-ended questions indicated that coping with discrimination was a major aspect of coming to terms with their racial/ethnic identity. A majority (78%) reported that they had either considered themselves to be white or wanted to be white when they were children [45].

Song and Lee [46] examined ethnic identity and cultural socialisation among 67 Korean-born adopted adults. Questions focused on four developmental periods: childhood, adolescence, young adulthood, and adulthood. Seven categories of cultural socialisation were identified in the qualitative data, including three categories which showed a positive correlation with ethnic identity: (1) exposure to or experience of multicultural diversity, such as living in diverse, heterogeneous communities, (2) developing an awareness of what it means to be a racial minority and an adopted person, and (3) visiting Korea and searching for one's birth/foster families. A significant positive correlation was found between cultural activities experienced during young adulthood (ages from 18 to 21 years) and ethnic identity [46]. Participants showed an increasing interest over time in “lived experience” activities such as socialising with Korean people or visiting Korea as they entered adulthood, as opposed to cultural activities such as eating Korean food or learning a Korean martial art [46]. This echoed findings from McGinnis et al. [45].

Another US-based survey of 78 internationally adopted adults (mean age, 29 years) explored self-esteem, ethnic identity, and cultural socialisation [47]. Again, several significant correlations were identified. Regression analysis identified that belongingness and marginality mediated the relationship between cultural socialisation and self-esteem. In other words, the adopted people's self-esteem was linked to* “a feeling that they belonged to their adoptive family as well as believing that they are not marginal in the majority culture, both of these qualities arising from the opportunities to get involved with their birth culture”* ([47], p. 167).

In the British Chinese Adoption Study, three indices were developed to assess the extent of connectedness to Chinese communities and culture, connectedness to UK society, and self-regard in relation to Chinese appearance [6]. The scores from these indices were then examined alongside interview data that explored these areas plus experiences of prejudice and racism. The women generally saw themselves as both British (via nationality and cultural socialisation) and Chinese (by genetic inheritance). However there were those for whom this question was difficult or preoccupying. Thirty-two percent were identified as having “some connection” to Chinese communities and/or culture using the first index. Active interest in the Chinese communities and culture was not associated, as may have been predicted, with indicators of better mental health [6]. Although the study did not find any associations between connectedness (or lack thereof) to Chinese culture and communities and the mental health measures, this does not discount the fact that, for some women, working out complex issues of identities had proved to be difficult [6].

#### 3.3.3. Dealing with Other People's Reactions, Degrading Attitudes, and Racism

Findings from three qualitative, interview-based studies were reported in the four papers listed in Table 2 [32, 33, 42, 48]. All explored variations on a theme of dealing with other people's reactions and attitudes. Docan-Morgan [32, 33] examined the experiences of 34 adults (mean age, 26 years) adopted from South Korea to the US by white adoptive parents. Nearly all participants (*n* = 31 of 34) could recall examples of intrusive interactions, although almost a third (*n* = 10) reported such incidents to be rare. Five types of interactions were identified: relational comments/questions (such as “Is she your real sister?”), compliments (“Asian babies are so cute!”), stares, mistaken identities (such as a brother being mistaken for his adoptive sister's boyfriend), and “adoptee-only” interactions (comments made only to the adopted person, not within earshot of family members).

In a separate paper, Docan-Morgan [33] reported on participants' experiences of “racial derogation.” Three main types of racial derogation were identified from the interview and survey data: appearance attacks (comments or gestures mocking their physical appearance), perceived ethnicity attacks (ethnic stereotyping such as racist name-calling or gestures such as mock karate), and physical attacks (including throwing of rocks, being tripped up, or fighting).

Lindblad and Signell [42], in interviews with 17 Swedish internationally adopted women, explored the women's experiences of and reactions to degrading attitudes towards them on the basis of their Asian appearance and being adopted. Different types of degrading sexual attitudes were identified, for example, comments about their sexual availability or assumptions about their relationships (such as being “paid” to be someone's partner, either as a sex worker or a bride). Nonsexual comments were no less tiresome, such as being called racist names or general hostility expressed towards immigrants. Negative adoption-related attitudes fell into two categories: (1) implying that the women should feel “eternally grateful” to their adoptive parents or (2) asking overly intrusive questions about their origins and families. The women's reactions and strategies to dealing with such incidents changed over time: childhood embarrassment turned to anger, sadness, and, occasionally, fear in later years [42]. Although most women had someone they could confide in, confidantes' reactions varied from supportive to disbelieving [42].

In another study from Sweden that focused on “everyday racism and ethnic identity,” 20 internationally adopted adults described day-to-day experiences of discrimination, for example, being followed in shops or facing heavy scrutiny at border and customs controls in comparison to their white Swedish peers [48]. Participants' views varied; some put questions about their origins down to “mere curiosity,” while others reported feeling regularly harassed. The authors emphasised that participants felt they had to “explain” themselves to other people. This finding chimed with evidence from other studies [6, 32].

In the British Chinese Adoption Study, most of the women had been on the receiving end of racism or prejudice in some form. This ranged from being called names (as adults, not just in childhood) and discrimination at work to, at the extreme end, being physically attacked. Not all the perpetrators were white nor did all experiences take place in the UK [6]. Prevalence of and reactions to such experiences were notably varied across this group, who were a followed up cohort as opposed to a convenience sample. Some women reported such incidents to be rare and to have little bearing on their overall assessment of their lives; for other women, dealing with other people's attitudes was, at times, an on-going preoccupation. The experiences reported in the qualitative studies reviewed here were familiar with some of the BCAS interviews.

## 4. Discussion and Conclusions

This review gathers together the adult midlife studies following international adoption that fitted our previously stated inclusion criteria. It explores the merits of these studies to identify what progress has been made in answering questions about long-term outcomes.

The studies we have reviewed suffer some shortcomings that are impossible to overcome in understanding correlations between early experiences and adulthood, for example, lack of information on the birth parents of abandoned children or lack of preadoption information in many cases. Other deficits are endemic to adoption research, for example, isolating the contribution of adoption* per se*, amongst many other influences, to outcome. Not all studies had access to or collected data on all of our areas of interest: orphanage care, intercountry adoptive experiences, postadoption life events, and midlife outcomes. We are, therefore, some way from unravelling the links between such factors as influences on adult functioning in midlife.

As documented, a number of limitations restrict definite conclusions. Disadvantages exist in studies that use such different approaches to measurement within a limited body of empirical research. But while this creates difficulties in drawing out strong consistent messages, it also highlights the complexity involved in conceptualising and measuring adult lives with atypical histories [49]. As the field develops, we would encourage researchers to use or develop tools whose theoretical origin is made explicit, which are conceptually clear, suited to this specific population, and widely available.

In addition to differences in research design and loss to follow-up, the samples themselves differ in important ways. Cohort effects may be important in influencing outcome, given that the children in the studies were born and placed at different points in time, and therefore many factors such as adoption procedures and medical and social support services, as well as social attitudes, are likely to have varied. Some studies involved only ex-orphanage samples, while others included some children whose early experiences were in foster care or were unknown. Children from some regions of origin, such as Eastern Europe, may be at greater risks for poorer outcomes, but the sampling strategies did not always lend themselves to testing for differences on this basis. Homogenous samples, or samples that are clearly disaggregated, lead to more easily interpretable and confident conclusions. Similarly, the availability and selection of appropriate comparison groups are vital to ensure that any differences in outcome neither are overlooked nor are due to other differences between samples.

Nevertheless, some tentative directions of travel within the body of research are detectable. Psychological functioning was problematic for a subsample in all those studies where this was measured, and in two studies this was significantly worse than comparison groups. While the evidence of elevated risks of severe mental health problems is limited to a minority of the internationally adopted groups in all of the studies discussed here, it cannot be dismissed. As with younger cohorts, therefore, enduring effects were found in some cases, although the extent, domains, and proportion affected vary across studies.

In studies that have found significant raised risks for infrequent behaviours such as suicide or very poor mental health (in both adulthood and younger life phases), closer examination of the data serves as a reminder that, overall, the proportion of internationally adopted people with such severe outcomes is a small minority. Even a small increase in group-level risk signifies a greater number of individuals and families who have likely experienced enormous distress and pain. However, caution must be exercised in extrapolating findings. While acknowledging the seriousness of such results, it is important to ensure that they are not interpreted in a way that suggests that a majority of internationally adopted people experience such pronounced difficulties. Article titles and abstracts, which cover only brief “headlines,” can obscure some of the complexities of how increased risks operate across the sample. Effect sizes and amount of variance may be more important than *p* values: for example, statistically significant results may be identified, but with a small effect size.

Conversely, those studies that focus on the extreme end of mental health outcomes may miss more subtle or internal psychological challenges, such as those relating to origins, identities, and how people locate themselves socially. Research based on smaller interview or self-report questionnaire data collected on these issues has started to put flesh on the bones and has shown a notable replication of patterns across samples in different countries. Studies focused on the prevalence of poor mental health and the related risk and protective factors must take account of these findings.

As mentioned earlier, this review was conducted in the context of the authors' involvement in the British Chinese Adoption Study; it identified midlife outcomes that were better than predicted. Despite early years in orphanage care, followed by international adoption, this did not appear to raise the risk of poor outcome. These of course were by no means extremely depriving institutions, and the adoptive homes were largely, although not always, supportive, consistent, and loving. As a group, the women's lives at nearly 50 were reported as mainly positive: they had mostly led settled lives, with good educational and career achievements. Many had enjoyed becoming parents themselves. Well-being and life satisfaction scores were commensurate with those of a large, sex- and age-matched, UK comparison group. However, a retrospectively reported poorer quality adoptive home was associated with poorer outcome. The knowledge of an atypical start to life and the experience of feeling different in appearance and being treated differently were a lifelong preoccupation for some (to a greater or lesser extent for individuals) but, as a group, this did not appear to have compromised their current psychological and social status [5].

### 4.1. Strengths and Limitations of This Review

In addition to the strengths and limitations of each study, this review also had its own benefits and restrictions. By focusing on midlife, we excluded several studies from early adulthood that may contain important messages for research with older cohorts. However, by including quantitative, qualitative, and mixed methods studies, a broader range of potential influences that should be considered in future research designs were revealed.

In searching the relevant literature, this review has been less able to address certain questions. With a lack of specific, reliable, and contemporaneous information on early experiences, it was often hard to trace links with possible long-standing difficulties. Furthermore, the investigative methods employed in most studies have yet to capture the internal conflicts and preoccupations of adopted people, which may be as revealing an aspect of outcome as mental health scores. Finally, data on the environments and experiences that may have contributed to recovery following adversity are less available, in particular on how changes in life stresses in adult life may interact with possible underlying vulnerabilities.

### 4.2. Implications for Future Research

Although comparisons across standard measures are essential to understanding any lingering risks or continued recovery from early events, we need to know more about the life experiences of these populations and changes over time. As Rushton [49] point out, the measures need to be more adoption relevant. GHQ scores alone, or similar mental health checklists, might give a helpful snapshot in relation to other groups where the same tools are employed but are not going to reveal much about adopted people specifically.

We are still some way from identifying, of all the myriad possible influences, why in most cohort studies a subsample does substantially worse. Research from younger groups suggests that age at placement and quality of care, or some combination of the two, can influence outcomes in middle and late childhood or even early adulthood. This is less clear in later adult outcome studies, because either such data are missing or age at placement has not been found to predict outcomes consistently. In the two studies of groups from psychosocially depriving, rather than globally depriving, orphanage care, little or no evidence was found for increased risks for poor psychological adjustment in adulthood.

Part of the challenge lies in the number of areas that make up a life in midadulthood. As age increases beyond the early twenties, so does the number of participants likely to have experiences such as parenthood, one or more partnerships, changes in other relationships, dealing with bereavement of major figures (parents, friends, or even partners/children), fluctuations in health, or triumphs and stresses related to work or education. Thus examining of adult lives involves a potentially greater number of “life experience” domain influences than research with younger cohorts. The British Chinese Adoption Study research is continuing in order to evaluate the relative contribution that emerging adulthood and midlife events and experiences make to outcomes.

Clearly, all methods bring some benefits at the expense of losing other possibilities; this is not unique to the topic of international adoption. But in an area with relatively limited literature, it is important that all studies, regardless of their own methodological approach, build on research from across the field. We have paid attention to the findings from both large-scale quantitative research and smaller interview-based studies because we believe that learning across the methodological spectrum is the best way forward in developing a strong evidence base. For example, data from smaller interview-based studies may suggest previously unidentified but potentially important influences on outcomes, which can then be tested for replication across larger cohorts.

Further research needs to be conducted to see whether findings from individual studies are replicated across different groups. In those studies indicating elevated risks, the key factors and likely pathways leading to this outcome need to be revealed. Furthermore, studies that enable testing of whether such differences diminish over time are required. The publication of results from subsequent follow-ups of existing adoption cohorts, as well as new prospective longitudinal studies using more comprehensive data, may be able to answer some of these questions (see [50]). If longitudinal studies of internationally adopted cohorts who are now in adolescence or early adulthood [51, 52] continue on to midlife, they will offer rich longitudinal data for exploring how internationally adopted people's lives change over time. At a later stage, studies with younger cohorts, which have been able to collect primary data from the orphanages, will add extra dimensions to the picture [53, 54].

Most adulthood studies of international adoption conducted to date have used self-reported data from participants or data from national records. Some potentially illuminating approaches have yet to be employed, for example, gaining the views of people close to adopted adults, such as a partner or close friend, to compare them with self-reported or other data. Missing completely from the current picture are studies that compare the experiences and outcomes of those who stayed in orphanage care versus those who were internationally adopted or cross-national comparisons of internationally adopted adults raised in different countries. In addition, some important areas such as adult attachment, or research designs that enable exploration of mechanisms underlying associations between early experiences and adult outcomes, have yet to be tackled more than once. Over time, it is to be hoped that opportunities for studies with a greater variety of complementary research designs will increase.

Overall, there is a somewhat narrow focus on individual psychology in studies to date. But national, societal, and cultural context matters. While it may be obvious that individuals who grow up in the UK, for example, are likely to have different experiences from those brought up in the US or Scandinavia, even within one country contexts can vary greatly: compare, for example, New York City and rural Texas. Differences may relate to policy and practice, attitudes towards international adoption and “families of difference,” local histories of race relations, and levels and types of race-based mistreatment. Events as diverse as a change in government, a celebrity adopting a child from overseas, or a high-profile child protection case can all affect public perceptions and have a knock-on effect on the experiences of internationally adopted people and their families.

Our general conclusion is that longer-term follow-ups of internationally adopted people have much to offer in refining models in developmental psychology. In particular they may help to fill out the picture between early care and midlife with greater recognition of the potentially restorative influence of a stable substitute family environment and the contribution made via their adult lives and experiences.

## Figures and Tables

**Table 1:** Studies with across-group comparisons.

Author(s) and year of publication	Sample size and characteristics	Preadoption circumstances	Age of participants^1^	Methods incl. main measures/interview topics	Comparison group	Main findings
Irhammar and Bengtsson, 2004 [40]	*N* = 40. All adopted between 1970 and 1977 and brought up by families in southern Sweden. Two-thirds were females. This group was formed of the oldest participants from previous study of 152 adolescents.	88% born in Asia (mostly India, Thailand, and Sri Lanka), and the remainder mostly in Latin America. All except one had spent some time in orphanage care prior to adoption.	Mean age: 28 years; range: 25–34 years.	Measures included Adult Attachment Interview (AAI, semistructured), mainly about relationships with adoptive parents during childhood. Transcripts coded to adult attachment classifications: autonomous, dismissive, or preoccupied.	Compared to AAI results of a “norm group” of nonclinical mothers from a meta-analysis.	Adopted group's AAI scores were not found to differ significantly from nonclinical comparison group. Revisiting country of origin and ethnic self-identity were not related to attachment classification, but later age at adoption predicted greater likelihood of dismissive or preoccupied attachment classification.

von Borczykowski et al., 2006 [35]	*N* = 6,065. Born between 1963 and 1973. Two-thirds females. Age at immigration available for 90% of the sample, of which 54% <2 yrs; 26% 2-3 yrs; and 20% >4 yrs. Identified via National Swedish Registers. Main areas of origin were Far East Asia (54%) and South Asia (18%).	No specific information on this cohort, general discussion of possible preadoption adversity.	Range: 29–39 years.	Suicide attempt and suicide death data taken from national hospital discharge and cause of death registers.	(1) 7,340 Swedish-born age-matched domestically adopted adults. (2) 1,269,318 age-matched nonadopted Swedish-born adults. (3) 3,616 siblings (adopters' birth children).	IC adopted group had higher suicide attempt (RR 4.5) and suicide death (RR 3.6) rates than gen. pop. and sibling samples. IC adopted women's risk compared to other female groups was elevated to a greater extent than men's. Domestically adopted adults also had higher risks than gen. pop. but less than the IC adopted.

Tieman et al., 2005 (a), 2006 (b), and 2008 (c) [25–27]	(a) *N* = 1,484. (b), (c) *N* = 1,417. Adopted into Holland 1972–1975 at mean age of 28 months. 55% females. Accessed through central records of adoption (sample (a) = 72.5% of original sample at earlier sweep). Attrition rate higher for men with higher scores (therefore more problems) on Child Behaviour Checklist.	Approx. 47% experienced neglect and 12% preadoption abuse. Rates based on data reported by adopters, using predefined scales of level of adversity; only information that parents reported as being “certain” of was included.	Range: 22–32 years at most recent time of data collection.	(a) Composite International Diagnostic Interview (standardised psychiatric interview that generates DSM-IV diagnoses). Some items from National Institute of Mental Health Diagnostic I/v Schedule. (b) Social functioning assessed via standardised semistructured 115-item interview. (c) Measures included: questions about searching for birth parents; DSM-IV diagnoses (see above); Child Behaviour Checklist; family functioning.	(a) 695 age-matched general population sample. (b) Subsample of 713 adults from the above gen. pop. sample, on whom additional data was available. (c) Within-group only.	(a) Adopted group at higher risk than control group to meet criteria for anxiety disorder (1.52 times), substance abuse or dependence (2.05 times), and, for men only, mood disorder (3.76 times). No significant difference for disruptive disorder, which differs from findings from earlier follow-up with same cohort. (b) Adopted group less likely than nonadopted to be living with a partner or have had a relationship lasting more than a year. But adopted people without partners functioned better than their nonadopted single counterparts. Authors conclude that groups were similar in social contacts. (c) 32% had searched for birth parents. Greater likelihood to search was associated with country of origin, older age at placement, greater interest in searching during adolescence, more problematic behaviour, and psychiatric diagnoses. Greater interest in (but not levels of) searching found for females.

van den Berg et al., 2008 [28] (same sample as Tieman et al. studies)	*N* = 1,475	As above.	As above.	Adopted adults' self-reported ratings of internalising and externalising problems on standardised scales. Parented-reported Young Adult Behaviour Checklist.	Within-group only: compared biologically related siblings, nonrelated siblings, and singles.	Only study to explore genetic and environmental influences on adult outcomes. Both data sources (self- and parent-report) indicated that genetic influences were greater for internalising problems and environmental influences were greater for externalising problems. This reversed findings from the same sample during adolescence.

van der Vegt et al., 2009 (a), 2009 (b), and 2010 (c) [29–31] (same sample as Tieman et al. studies)	(a) *N* = 1,364. (b) *N* = 623. (c) *N* = 429. (b) and (c) both subsamples from (a). Sample (a) = 64% of baseline sample. As above, selective attrition at follow-up was noted; nonparticipants had higher levels of mental health problems in childhood and adolescence.	As above.	(a) Mean age: 26.3 years; range: 22–32 years; SD: 1.4. (b) and (c) both subsamples from (a).	(a) Measures included interviews generating information on DSM-IV codes of mental disorders. Recorded these diagnoses individually plus created an “any disorder” variable. Plus parent reports on early abuse, neglect, and multiple preadoption placements. (b) Parent reports on preadoption adversity (as above). Cortisol levels collected 4 times per day via saliva samples. (c) As above, plus Child Behaviour Checklist.	Within-group only.	(a) Multiple early adversities associated with increased risk of adulthood anxiety (OR = 2.22; 95% CI 1.11–4.45); mood disorders (OR = 2.20; 95% CI 1.00–4.86); or substance abuse or dependence (OR = 3.81; 95% CI 1.62–8.98). After controlling for childhood onset of mental health problems, differences remained. Level of de novo onset suggests that consequences of early adversity can appear many years later. (b) Early neglect and abuse both predicted altered cortisol levels compared to nonneglected/nonabused groups. (c) Severe early maltreatment found to modify the relationship between anxiety disorders and cortisol secretion, but not mood disorders and cortisol secretion.

McGinnis et al., 2009 [45]	*N* = 179. Korean-born adults adopted into white US families. 18% men, 82% women.	Majority of time prior to adoption spent in orphanages (35%), foster families (39%), birth family (13%), unknown (11%), or other (2%).	Mean age: 31 years. All over 18 years.	Online questionnaire, incl. Family of Origin Scale, Multigroup Ethnic Identity Measure, Rosenberg Self-Esteem, and Satisfaction with Life. Also questions about (1) changes in self-identification and (2) support services.	156 white American-born adopted adults, from same total sample as Korean-born group. Mean age: 44 years (13 years older than Korean-born group).	Adoption is an increasingly significant aspect of identity across lifespan for both groups; “race”/ethnicity is increasingly important for Korean group, peaking in adulthood. Feeling more comfortable with adoptive identity was associated with higher life satisfaction for White group. In contrast, feeling more comfortable with adoptive identity was associated with female gender, higher life satisfaction and higher self-esteem for Korean-born group.

Storsbergen et al., 2010 [36]	*N* = 53. Children adopted in infancy from Greece into Netherlands before 1970. 30 men and 23 women (of 60 randomly selected from 121 traced; total potential sample was 400).	Authors characterise this as a group who did not suffer severe deprivation: relatively limited number of caregivers in relatively limited number of caregivers in orphanage and attempts at consistency in care.	Mean age: 29 years; range: 25–36 years.	General questionnaire about adult life circumstances and adoption, plus mental health (Symptom Check-List 90), well-being (Satisfaction with Life scale), and self-esteem (Rosenberg).	Normative data on Dutch-born young adults, taken from different sources for each measure.	Only significant difference from comparisons on mental health, well-being, and self-esteem was higher rate of depression for adopted men in comparison to nonadopted men. Within-group: those who searched for birth parents reported more difficulties in mental health, well-being, and self-esteem. Further analysis identified that negative appraisal of adoption was a stronger predictor than search status for mental health outcomes and well-being.

Rushton et al., 2012 (a) and 2013 (b) [5, 6]	*N* = 72. Hong Kong ex-orphanage girls adopted into the UK in the 1960s. Duration of orphanage care: mean age: 20 months (range: 5–83 months; SD: 13.5). Age at placement for adoption: mean age: 23 months (range: 8–83 months; SD: 14) Participants were from original groups of 100 women traced individually nearly 50 years later and recruited to the study.	Relatively well-run orphanages with adequate physical care and nutrition but overcrowding and lack of opportunity for selective attachment to adult carers.	Mean age: 48 years; range: 42–53 years; SD: 2.4.	Orphanage/adoption records. Questionnaires covering mental health (General Health Questionnaire and Malaise Inventory), self-esteem (Rosenberg), life satisfaction, personality profiles, community connectedness, partnerships, and adoptive family relationships. Interviews on life history and current circumstances (usually 2-3 hours).	(a) Within-group only. (b) 5,115 age- and sex-matched general population sample (National Child Development Study) and a domestically adopted subsample (*n* = 50) drawn from the same dataset.	(a) Ethnic and social identification not found to predict psychological adjustment. (b) Outcomes were commensurate with the comparison groups in terms of mental and physical health measures. Serious psychiatric and social difficulties were largely absent. Timing and extent of exposure to orphanage care did not influence outcome, but self-reported poorer quality adoptive experience and a negative view of their adoption were significantly associated with poorer mental health outcomes (difference in means = 0.76, 95% CI 1.33–0.19, *p* = .01; difference in means = 1.2, 95% CI 0.68–1.73, *p* = .01, resp.).

^1^Age of participants at point of data reported in this paper. IC = intercountry, OR = odds ratio, RR = risk ratio, gen pop = general population, and I/v = interview.

**Table 2:** Single-cohort studies.

Author(s) and year of publication	Sample size and characteristics	Preadoption circumstances	Age of participants^1^	Topics/questions covered	Mode of analysis	Main findings
Mohanty et al., 2006 [47]	*N* = 78 79% of participants from US, the remainder from “other Western countries.” Adopted from Korea (60%), Vietnam (23%), and elsewhere. Median age at adoption: 10 months (range: 1–156 months).	67% in orphanage care, 22% foster care, 5% multiple placements, and 5% other settings.	Mean age: 29 years, range: 18–44 years, and SD: 5.96.	Web-based survey including self-esteem measure (Rosenberg) and ethnic identity and cultural socialisation scales developed by the research team.	Statistical analysis, using path analysis models.	Most reported little cultural socialization and not growing up in areas “with neighbours who reflect my race.” Cultural socialisation and self-esteem both correlated positively with feelings of “belonging” within adoptive family and negatively with feelings of “marginality” in majority culture. Belonging and marginality mediated relationship between cultural socialisation and self-esteem.

Docan-Morgan, 2010 and 2011 [32, 33]	*N* = 34 26 women, 8 men. All adopted from Korea into white US families. 53% had Korean-born adoptive siblings. Recruited via Asian American university clubs and Int'l Korean Adoptee Gathering 2007. 34 adopted adults (23 interviews and 11 via online survey).	No information given about preadoption experiences, except in passing in vignettes from interview reports.	Mean age: 26 years, range: 18–40 years, and SD: 6.6.	Qualitative interviews/online survey: questions about intrusive interactions from strangers (e.g., excessive personal questions and being stared at when with adoptive family members) and dealing with race-based mistreatment.	Thematic analysis of interview data then survey data. Member-checking by sending initial research paper to participants.	Participants reported frustration and defensiveness as a result of obvious intrusions, such as strangers' stares or comments about not being a “real” family, but also with excessive compliments (“Asian babies are so cute!”). Racist experiences included name-calling, appearance mocking, stereotyping, and physical attacks. Range of adoptive parents' reactions described (defiance; using humour; displaying pride in family). Not all participants confided in parents.

Tigervall and Hübinette, 2010 [48]	*N* = 20 Adopted from Korea (*n* = 10) and other countries (*n* = 10). Gender distribution not given. (Interviews were also conducted with eight nonrelated adoptive parents; results not described here.)	No information given.	Range: 21–48 years (mean age and SD not given).	Semistructured interviews about experiences of race-based mistreatment, including discrimination and exclusion.	Thematic analysis based on “social-constructivist” concepts (i.e., emphasis on concepts such as “race” as relational and identities as fluid and negotiable).	Day-to-day experiences of discrimination described, such as being followed in shops or facing heavy scrutiny at border/customs controls in comparison to white Swedish peers. Also regularly facing questions about their family and origins. Participants' views varied; some saw questions as “mere curiosity”; others felt regularly harassed.

Lindblad and Signell, 2008 [42]	*N* = 17 Women adopted from South Korea (*n* = 15) and Thailand (2); all raised in Sweden.	Age at adoption ranged from a few months to 4 years; most (*n* = 10) adopted within first year.	Range: 18–35 years; half the group were aged over 30 years.	Interviews focused on “experiences of degrading attitudes with probable relation to Asian appearance”; the perpetrators; subjective reactions and strategies for coping with such attitudes; communication about them.	Open coding based on grounded theory. Final categorisation agreed upon following extensive dialogue and discussion between the two authors.	Degrading attitudes were reported in relation to both Asian appearance and adoption, including comments about their perceived sexual availability/libido or general hostility expressed towards immigrants. Perpetrators came from all age groups, and events were reported as repeated experiences, not one-off events. These experiences provoked a wide range of feelings, from anger to sadness.

Song and Lee, 2009 [46]	*N* = 67 55 female, 11 male, and one unidentified. Korean American adopted adults recruited via adoption agencies, conferences, and snowballing. Described as generally highly educated (61% had completed undergraduate degree or higher).	Mean age at adoption 22 months (range: 0–195 months; SD: 29.46). 60% had one and 28% had two preadoption placements (proportion of orphanage versus other placements not specified).	Mean age: 27 years, range: 18–49 years, and SD: 6.6.	Survey included Multigroup Ethnic Identity Measure [55] and open-ended questions about cultural socialisation in childhood, adolescence, and adulthood, including adoptive parents' strategies and participants' own interest/effort.	Thematic analysis of qualitative responses. Resulting categories were then compared with MEIM scores to identify correlation between cultural socialisation and ethnic identity.	Factors that correlated positively with ethnic identity were living in multicultural community, racial awareness (developing an awareness of being part of racial minority group and an adopted person), and visiting Korea/searching for birth/foster family. Significant positive correlation between cultural activities during ages 18–21 years and ethnic identity. Increasing interest over time in “lived experience,” for example, visiting Korea and socialising with Korean people.

^1^Age of participants at point of data reported in this paper.
